# Optimization of soaking conditions (temperature and time) on physicochemical properties of selected parboiled rice varieties grown in Eastern Ethiopia

**DOI:** 10.1002/fsn3.3298

**Published:** 2023-03-13

**Authors:** Ziad Ahmed, Sirawdink Fikreyesus Forsido, Chala G. Kuyu, Ebisa Olika Keyata

**Affiliations:** ^1^ Department of Food Science and Nutrition Jigjiga University Jigjiga Ethiopia; ^2^ Department of Postharvest Management Jimma University Jimma Ethiopia; ^3^ Department of Food and Nutritional Science Wollega University Shambu Ethiopia

**Keywords:** cooking time, optimization, *Oryza sativa*, parboiling, physicochemical properties

## Abstract

This study aimed to optimize soaking temperature and time for better physicochemical properties of parboiled rice varieties grown in Eastern Ethiopia. Two brown rice varieties (NERICA‐4 and NERICA‐6) were collected from the Somali Regional Agricultural and Pastoral Research Center in Gode. The experiment was designed to aid the design expert software using box–behnken experimental design of response surface methodology to optimize the effects of soaking temperature (60–70°C) and soaking time (4–6 h). Relevant physical and chemical composition properties of the parboiled rice varieties were investigated using standard methods. Numerical optimization of the responses was performed using design expert software. The results showed that soaking time and temperature significantly (*p* < .05) influenced the physicochemical quality of studied brown rice varieties. The optimal soaking temperature and time were 65°C and 6 h, respectively, for NERICA‐4. Under these conditions, the optimum response variables obtained were 375.37 N, 52 min, 12.3%, 1.24%, 13.86%, 2.17%, 3.2942%, 67.1171%, 343.5 kcal/100 g, 274.72 mg/100 g, 318.35 mg/100 g, and 268.31 mg/100 g for hardness, cooking time, moisture, ash, protein, fat, fiber, carbohydrate, energy, magnesium, and potassium and phosphorous content, respectively. However, 65°C and 5 h were optimum soaking temperatures and time for NERICA‐6, giving hardness, cooking time, moisture, ash, protein, fat, fiber, carbohydrate, energy content, magnesium, potassium, and phosphorous of 375.18 N, 52 min, 12.2%, 1.4%, 11.54%, 2.29%, 2.89%, 69.6%, 345.42 kcal/100 g, 156 mg/100 g, 105.9 mg/100 g and 136.9 mg/100 g, respectively. The findings showed that rice varieties, in particular NARICA 4, were processed under optimal parboiling conditions in the study setting for better physical properties, proximate composition, and mineral content.

## Graphical abstract

Soaking time and temperature significantly affected the physicochemical properties of both rice varieties. The lower cooking time between 60 and 62°C of soaking temperature for 4 h. Numerical optimization results generated better rice in terms of physicochemical properties.

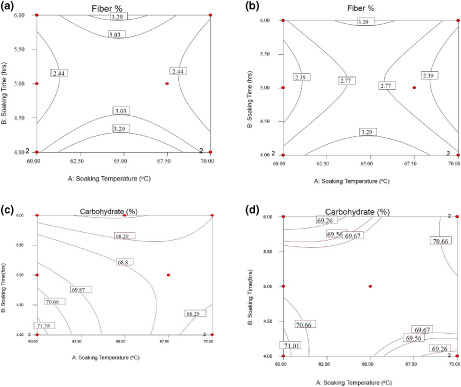

## 1 INTRODUCTION

Rice (*Oryza sativa* L.) is an essential staple crop and the fastest‐growing food product in Sub‐Saharan Africa (Alemu & Thompson, 2020). Among the different rice varieties grown in the world, brown rice is a nutrient‐dense food high amount of fiber and a good source of magnesium, phosphorus, potassium, thiamine, niacin, vitamin B6, and an excellent source of manganese (Rathna et al., 2019).

According to the Central Statistical Agency (CSA, 2013) of Ethiopia report, Approximately 115 thousand farmers were involved in the cultivation of rice in 2012/13 and that number rose to about 161 thousand in 2017/18. Similarly, the area covered grew from around 41,000 ha in 2012/13 to approximately 53,000 ha in 2017/18 (CSA, 2018), and output climbed from approximately 121,000 tons in 2012/13 (CSA, 2013) to 151,000 tons in 2017/18 (CSA, 2018). Despite an increase in productivity over time, grinding the rice results in a significant postharvest loss. Ethiopia's rice production has steadily increased from time to time. For example, rice output in Ethiopia was reported to be 139,780 tons (FAOSTAT, 2015) and 170,630 tons (FAOSTAT, 2019), respectively, on 45,454 ha and 57,576 ha of land. Regarding productivity, nutritional quality, cooking, and eating quality, rice grain quality remains a significant concern for rice breeders, farmers, and consumers (Custodio et al., 2019).

Regional and federal agricultural research centers, including Adet (national coordinator), Gonder, Bako, Bonga, Gambella, and Gode, are collaborating mainly in the national rice variety and adaptation trials, organized and coordinated by the national coordinating center. So far, variety development has been focusing exclusively on pure lines and is targeted to address mainly upland and lowland rain‐fed and, to some extent, irrigated ecosystems (Sewagegne, 2011). Ethiopia implemented NERICA varieties such as NERICA‐1, ‐2, ‐3, ‐4, ‐6, and Suparica‐1 in addition to local varieties such as X‐Jigna and others (Teshome & Dawit, 2007).

Among the rice mentioned above varieties, NERICA‐4 and NERICA‐6 were selected in this study due to their widely cultivated, well adapted to the environmental conditions, excellent quality and yield of head rice, not broken when de‐husked, they are more consumed by the local community and more commercialized than many varieties grown in the Somali regional agricultural and pastoral areas. Regarding utilization techniques, rice passes through a series of processing operations (de‐husking, parboiling, and milling) before consumption (Kale et al., 2017). Among unit operations, soaking and parboiling are essential steps influencing rice quality (Sareepuang et al., 2008). Knowledge about the effects of different unit operations on the characteristics of rice quality helps to make rice of better quality (Juliano & Tuaño, 2018). Parboiling is a process designed to improve the quality of rice and consists of soaking, steaming, and drying operations (Buggenhout et al., 2014). Parboiling is identified as a key technique to improve the physical properties, nutritional value, and cooking quality of rice (Meresa et al., 2020). Soaking is a hydration method in which water is absorbed through diffusion and then migrates into the rice kernel (Mir & Bosco, 2013).

In Ethiopia, most scientific information was published on the effect of parboiling conditions on rice's physical and cooking quality (Abera et al., 2021; Meresa et al., 2020). Nevertheless, there is a piece of limited scientific information on the optimization of conventional soaking time and temperature on the physicochemical properties of selected parboiled rice varieties (NERICA‐4 and NERICA‐6) grown in Eastern Ethiopia. If processing conditions are not optimized, physicochemical quality may be affected and result in inconsistent product quality. This variability manifests in the variation of specific quality parameters, such as the physical properties, particularly hardness and cooking time, and proximate and mineral composition of the final processed product.

Thus, optimization needs to produce consistent and high‐quality rice to support large‐scale commercial parboiled rice to compete with imported rice. In addition, developing an optimum parboiling method will enhance the widespread commercialization and use of products across the country. This, in turn, creates better market opportunities for rice farmers. Given this, this study aimed to optimize soaking conditions (temperature and time) during parboiling for better physical properties, proximate composition, and mineral content of selected rice varieties grown in the Somali Regional Agricultural and Pastoral Research Center in Gode, Ethiopia.

## 2 MATERIALS AND METHODS

### 2.1 Materials collection and sample preparation

Two rice varieties (NERICA‐4 and NERICA‐6) were collected from the Somali Regional Agricultural and Pastoral Research Center in Gode, Ethiopia in February 2020. About 10 kg of each variety of paddy rice was de‐husked using a rubber‐roll Sheller. The paddy rice is cleaned from foreign materials such as sand particles, stones, straws, seeds, and other impurities.

### 2.2 Experimental design

The experiment was designed to aid the design expert software (design expert® version 6.02, Minneapolis, USA) using box–behnken experimental design of response surface methodology with two numerical factors (soaking time [4–6 h] and soaking temperature [60–70°C]) and a categorical factor (rice variety NERICA‐4 and NERICA‐6) were used and generated 17 experimental points (Table 1). The minimum and maximum soaking time and temperature ranges were adjusted based on the recommendation of Heinemann et al. (2005) and Kale et al. (2015). Finally, physical properties, proximate composition, minerals content, and optimum soaking temperature and soaking time with better physicochemical properties were studied.

**TABLE 1:** Experimental runs generated using D‐optimal design for the coded value during parboiling of two rice varieties.

Std	Run	Coded value	Soaking temperature (°C)	Coded value	Soaking time (h)	Variety
11	1	−1	60.00	+1	6.00	NERICA‐4
17	2	−1	60.00	−1	4.00	NERICA‐4
1	3	+1	70.00	+	6.00	NERICA‐6
6	4	+1	70.00	−1	4.00	NERICA‐4
13	5	−1	60.00	0	5.00	NERICA‐6
7	6	−1	60.00	−1	4.00	NERICA‐4
4	7	+1	70.00	−1	4.00	NERICA‐6
14	8	+1	70.00	−1	4.00	NERICA‐6
16	9	+1	70.00	−1	4.00	NERICA‐4
9	10	−1	60.00	−1	4.00	NERICA‐6
5	11	−1	60.00	0	5.00	NERICA‐4
10	12	0	67.50	0	5.00	NERICA‐4
12	13	+1	70.00	+1	6.00	NERICA‐4
15	14	+1	70.00	+1	6.00	NERICA‐6
3	15	0	65.00	0	5.00	NERICA‐6
2	16	−1	60.00	+1	6.00	NERICA‐6
8	17	0	65.00	+1	6.00	NERICA‐4

*Note*: Y = β_0_ + β_1_A + β_2_B + β_3_C + β_11_A^2^ + β_22_B^2^ + β_33_C^2^ + β_12_AB + β_13_AC + β_23_BC.................Eq1 where Y = parameters, A = soaking temperature, B = soaking time, C = Soaking time.

### 2.3 Parboiling process

#### 2.3.1 Soaking

The soaking process was conducted according to the procedure described by Fan et al. (1999) and the treatment combinations generated by the design expert. Each treatment sample weighing 500 g of brown rice was soaked in a water bath (HH S4 water bath) all in a filter cloth immersed in hot water (60–70°C) for 4–6 h, followed by draining. The soaked brown rice was tempered at ambient temperature for 30 min.

#### 2.3.2 Drying

Drying is mainly conducted according to the procedure described by Zubair et al. (2015). Drying is done during the preparation of the paddy and after soaking and steaming before husking. The soaked paddy has to be steamed to attain about 30% moisture content wet basis. The soaked rice was dried by using the sun in the shade outdoors to dry the wet rice, which can be a multiple‐day process to reduce the moisture content between 10–14% for storage (Luh & Mickus, 1991) and the average time for drying soaked brown rice from 6 to 8 h and using of the sun drying for rice dependent on the weather conditions. After drying, samples were stored in airtight polyethylene bags for moisture equilibration and hardness stabilization.

### 2.4 Determination of proximate composition

The moisture content of the samples was investigated by using the convective oven drying method (105°C for 1 h) by taking about 3 g sample (dried sample powder) as described in the AOAC (2000) method 925.10. The micro‐Kjeldahl method determined crude protein content by taking about 1.0 g of the sample as described in AOAC (2000) method, 920.87. The crude fat content was determined by taking about 1.5 g of the sample by the Soxhlet extraction method using petroleum ether as a solvent (AOAC, 2000, method, 920.39). The crude fiber content was determined following AOAC (2000) method 962.09 after sequential digestion with 1.25% H_2_SO_4_ and 28% KOH, screened through 75 microns, drying, and ignition in a muffle furnace (Sx2‐4‐10, Zhejiang, China) to subtract ash from the crude fiber. The total ash content was determined gravimetrically after carbonizing about 2.0 g sample on a blue flame of Bunsen burner followed by ignition of the sample at 550°C until ashing was completed (AOAC, 2000, method 923.03). The difference is determined by total carbohydrate content (TCC) (FAO, 1998). The results of all proximate compositions were expressed in percentages (%). Energy value was calculated using Atwater's conversion ratios: 4 kcal/g for protein, 9 kcal/g for fat, and 4 kcal/g for carbohydrate (FAO, 2002). All the above‐indicated parameters were reported on a dry weight basis.

### 2.5 Determination of mineral content

The minerals content (magnesium, potassium, and phosphorous) were determined by atomic absorption spectrophotometer (AAS) following AOAC (2000) method 985.35 after carbonization on a heating plate and dry ashing of about 3.0 g of samples in a muffle furnace at 550°C until ashing was completed. The white ash was dissolved using 5 mL of 6 N HCl and dried on a hot plate, followed by the addition of 7 mL of 3 N HCl heating on a hot plate. Then finally, the solution was diluted to the mark (50 mL) with de‐ionized water.

### 2.6 Determination of physical properties

#### 2.6.1 Cooking time for parboiled rice

Cooking time of parboiled rice was determined according to the method described by Hasan et al. (2019). Five grams of parboiled rice for each treatment were weighed, decanted into 135 mL of vigorously boiling distilled water in a 400 mL beaker, covered with a watch glass, and then put into water boiled at 99.9°C in the water bath. After about 10 min of boiling, 10 grains were taken out from the boiling state one by one every minute with a perforated ladle. The grains were taken out immediately and pressed between two petri‐dishes to know whether it was well cooked. This was continued until at least nine out of the 10 grains no longer had opaque centers. The time taken for that to happen was recorded and considered the sample's optimum cooking time.

#### 2.6.2 Hardness of parboiled rice

The rice grain's hardness was measured using a texture analyzer (TA‐HD, Stable Micro Systems Ltd, Surrey, UK). A single compression force versus time program was used to compress single rice at a test speed of 0.10 mm/sec and return it to its original position. The actual clearance between the probe and the base in the instrument's load cell was fixed at 8 mm. When the probe moved down, it would compress the test sample kept horizontally on the base to a distance of 0.50 mm. The program was set to move the probe at 1.0 mm/min in both pretest and posttest phases.

### 2.7 Numerical optimization

Optimization determined the optimum soaking temperature and soaking time with better rice regarding physicochemical responses. Accordingly, a numerical optimization procedure was used for identifying the best conditions by incorporating appropriate constraints to establish the independent and dependent variables on the response of parboiling conditions. This context maximized ash, protein, fat, fiber, energy, magnesium, potassium, and phosphorous. In contrast carbohydrate, hardness, cooking time, and moisture contents were minimized.

### 2.8 Fitting the models

To check the adequacy of the models, a lack of fit test and regression coefficients were analyzed as a measure of the efficiency of a model to represent data in the experimental domain, at which points were not included in the regression (Montgomery, 2017). ANOVA results showed that the *R* ^2^ value of all dependent variables was greater than 0.80 indicating that the data explained a high proportion of variability. Moreover, for all analyzed data diagnostic tools like a normal plot of residuals were tested and indicated that the residuals of all parameters were normally distributed.

### 2.9 Statistical analysis

The data were analyzed and modeled using Design Expert® version 6.0.2, Minneapolis, USA. The essential terms in the models were identified by analysis of variance (ANOVA) for each response and accepted at a 0.05 level of probability. The regression coefficient (adjusted *R* ^2^) and lack of fit test checked the model adequacy. The soaking condition was optimized by setting target values for response variables (minimum, maximum, and range).

## 3 RESULTS AND DISCUSSION

### 3.1 Proximate composition and gross energy contents

Soaking temperature and time effects on proximate composition such as moisture, crude protein, crude fat, crude fiber, crude fat, total ash, total carbohydrate, and gross energy contents of parboiled rice varieties are presented in Table 2.

**TABLE 2:** Effects of soaking temperature and time on proximate compositions (%) and energy contents of parboiled rice varieties.

Tem (°C)	Time (h)	Varieties	Moisture %	Ash %	Protein %	Fat %	Fiber %	CHO %	Energy kcal/100 g
60	6	NERICA‐4	12.00	1.20	13.42	2.13	2.96	68.26	346.03
60	4	NERICA‐4	10.80	0.91	11.65	1.87	3.03	71.73	350.38
70	6	NERICA‐6	14.31	1.48	10.58	1.71	1.93	70.97	341.66
70	4	NERICA‐4	11.32	1.86	13.32	2.71	2.93	67.90	349.39
60	5	NERICA‐6	12.50	1.00	12.11	2.36	2.77	69.24	346.73
60	4	NERICA‐4	10.70	1.12	12.26	1.87	2.98	70.96	350.16
70	4	NERICA‐6	11.75	1.16	11.34	3.45	1.73	69.56	358.69
70	4	NERICA‐6	12.63	1.40	11.44	3.52	2.39	68.41	351.20
70	4	NERICA‐4	12.65	2.04	12.58	2.11	2.90	67.67	340.11
60	4	NERICA‐6	11.70	0.80	09.95	1.77	3.29	72.27	343.42
60	5	NERICA‐4	11.60	1.13	12.66	2.06	1.86	70.66	351.93
67.5	5	NERICA‐4	12.40	1.20	12.41	1.66	2.44	69.87	344.16
70	6	NERICA‐4	14.40	1.82	10.98	2.33	2.51	67.69	335.72
70	6	NERICA‐6	13.29	1.60	09.92	1.81	1.93	71.55	342.24
65	5	NERICA‐6	12.20	1.40	11.54	2.29	2.89	69.67	345.52
60	6	NERICA‐6	11.25	1.92	11.41	2.11	3.03	69.26	341.78
65	6	NERICA‐4	12.30	1.24	13.86	2.17	3.29	67.11	343.5

#### 3.1.1 Moisture content

**FIGURE 1:**
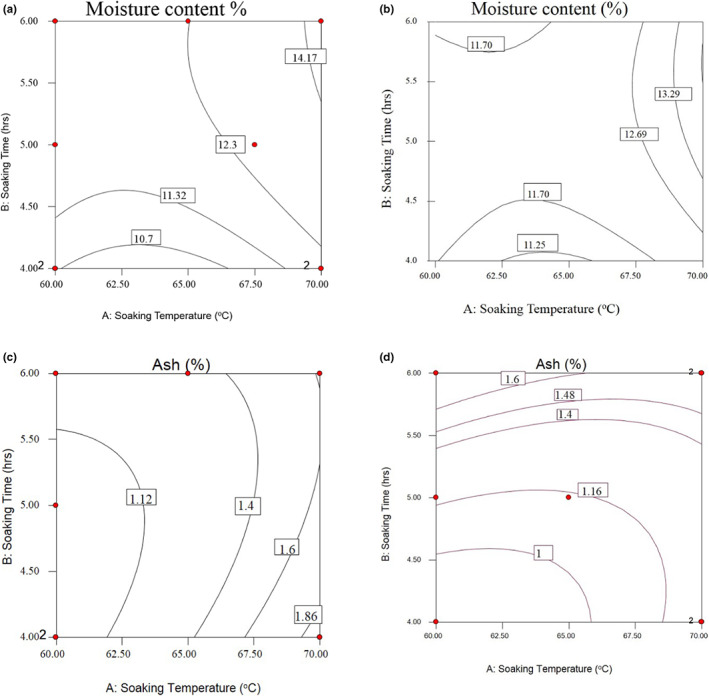
Response surfaces for moisture content of NERICA‐4 (a), moisture content of NERICA‐6 (b), ash content of NERICA‐4 (c), and ash content of NERICA‐6 (d) of parboiled rice kernels.

The mean moisture contents of parboiled rice varieties prepared at different soaking temperatures and time ranged from 10.70 to 14.40% (Table 2). The lower moisture content was recorded between 61 and 66°C of soaking temperatures at 4 h soaking time while the highest moisture content was recorded at 70°C soaking temperature and 6 h soaking time for NERICA‐4 variety (Figure 1a). For NERICA‐6, the lower moisture content was recorded between 62.5 and 66°C of soaking temperature at 4 h. The highest moisture content value was recorded between 68 and 70°C soaking temperature and 5.50–5.00 h soaking time (Figure 1b). The observed trend in the cooking time could be related to the effects of soaking temperature and soaking time. The value recorded in this study was similar to the result of Parnsakhorn and Noomhorm (2008). They indicated that the high moisture content could result from soaking time and storage humidity. Similarly, Roy et al. (2011) have reported that parboiled rice varieties showed an elevation in the amount of moisture content with an increase in soaking temperature that quickly passes through the bran layer of rice.

#### 3.1.2 Total ash content

**FIGURE 2:**
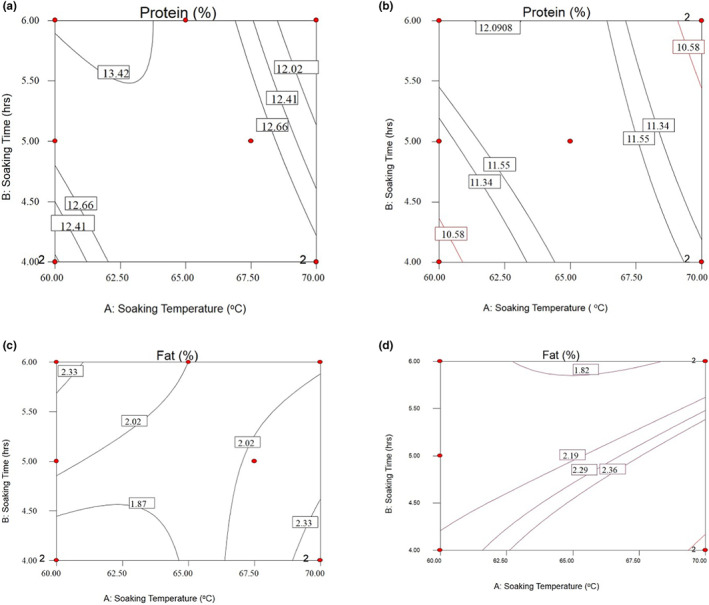
Response surfaces for the crude protein content of NERICA‐4 (a), the crude protein content of NERICA 6 (b), the crude fat content of NERICA 4 (c), and the crude fat content of NERICA 6 (d) of parboiled rice kernels.

As indicated in Figure 2c, the highest ash content for NERICA‐4 variety was recorded between 68 and 70°C of soaking temperature for 4 h of soaking times. The lowest ash content was recorded between 60°C of soaking temperature and at the 4.25–5.75 h of soaking time (Figure 1c). For NERICA‐6 variety, the highest ash content was recorded between 60 and 67°C of soaking temperature for 6 h. The lowest ash content was recorded between 60 and 66°C for 4–4.5 h of soaking time (Figure 2d). The difference could be due to the high temperature degrading the rice's other bio‐molecules and increasing the proportion of the minerals. Pathmanathapillai et al. (2016) stated that rice soaked at high temperatures during parboiling had the highest ash content than those treated with the lowest soaking temperature.

#### 3.1.3 Crude protein content

**FIGURE 3:**
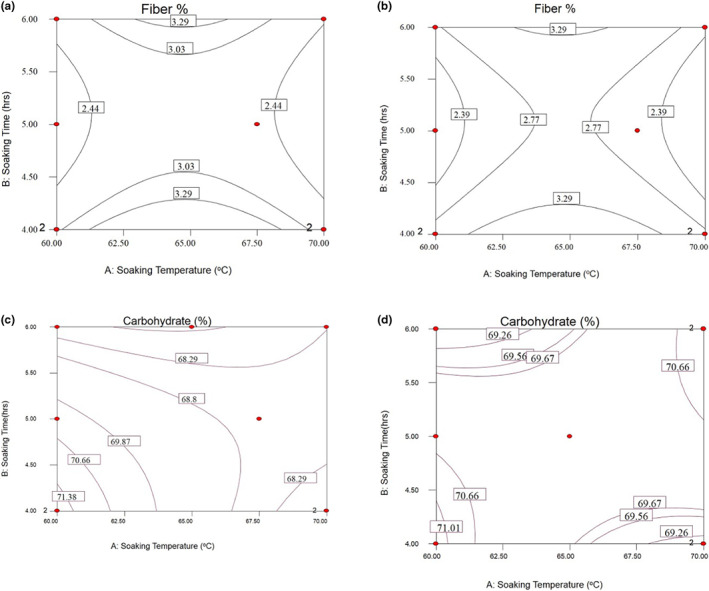
Response surfaces for the crude fiber content of NERICA‐4 (a), the crude fiber content of NERICA‐6 (b), the carbohydrate content of NERICA‐4 (c), and the carbohydrate content of NERICA‐6 (d) of parboiled rice kernel.

There were decreasing trends in protein content for both varieties with an increase in soaking temperature, as indicated in Figure 2a,b. For NERICA‐4 variety, the highest protein content was recorded between 61 and 62.5°C of soaking temperature for 6 h, whereas the lowest value was recorded between 67 and 70°C of soaking temperature for 4.5–6 h (Figure 3a). For NERICA‐6 variety, the highest protein content was recorded between 61.5 and 64°C of soaking temperature for 6 h, whereas the lowest protein contents value was recorded between 68.5 and 70°C for 5.5–6 h (Figure 3b). The findings similar to the results reported by Hasbullah et al. (2016) showed that soaking in 60°C hot water for 4 h resulted in the highest protein content.

#### 3.1.4 Crude fat content

Figure 2c shows that the highest fat content was obtained at a soaking temperature of 68.5–70°C for 4 h soaking times for the NERICA‐4 variety. In contrast, the lowest fat content was recorded between 60 and 64.5°C soaking temperatures for a 4–4.5 h of soaking time. For NERICA‐6 variety, the highest fat content was recorded between 62.5 and 70°C of soaking temperature for 6 h. The lowest fat content was recorded at a soaking temperature of 62.5 and 67.5°C for 6 h of soaking times (Figure 2d).

The variation observed in the response of fat content might be due to the migration of fat molecules from bran to endosperm at higher soaking temperatures and, as a result, enhanced fat extraction during analysis. This observation agrees with the work of Patindol et al. (2008). They reported that higher soaking temperature during parboiling enhances the extraction of fat from bran and lipid bound with other biomolecules like protein.

#### 3.1.5 Crude fiber content

**FIGURE 4:**
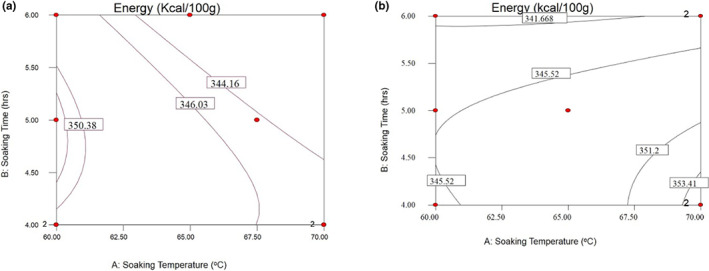
Response surfaces for the energy content of NERICA‐4 (a) and energy content of NERICA‐6 (b) of parboiled rice kernel.

Figure 3a indicates that the highest fiber content was recorded between 61.5 and 68°C of soaking temperature for 6 h. The lowest fiber content was recorded between 68.5 and 70°C of soaking temperature and 4–5 h of soaking time for NERICA‐4 variety. For NERICA‐6 variety, the highest fiber content was recorded between 62 and 69°C of soaking temperature for 6 h. The lowest fiber content was recorded at a soaking temperature of 69–70°C for 4–5.5 h (Figure 4b). The reported fiber pattern could be attributed to soaking temperature and soaking time. The soaking temperature was essential for the crude fiber content. The results presented in Figure 3a,b have shown that soaking temperature and soaking time decreased the parboiling rice's crude fiber content. This observation agrees with the work of Oko et al. (2012). They reported that higher soaking temperature and lower soaking time during parboiling lead to lower fiber removal from the bran.

#### 3.1.6 Total carbohydrate content

The highest carbohydrate content was recorded between 60 and 61.5°C of soaking temperature for 6 h for the NERICA‐4 variety. The lowest carbohydrate content was recorded between 62 and 66°C of soaking temperature and 4 h of soaking time (Figure 3c). For NERICA‐6 variety, the highest carbohydrate content value was recorded at 60.5°C of soaking temperature for 6 h. The lowest carbohydrate content was recorded between 60 and 63°C of soaking temperature and 6 h of soaking time (Figure 3c). The results showed that soaking temperature and soaking time decreased the quality of carbohydrates in the parboiled rice. The carbohydrate content increased with the decrease in soaking temperature and long/short time compared with non‐parboiled rice varieties. The observation was similar to the findings of Otegbayo et al. (2001). They observed that soaking temperature and a process that increases the carbohydrate content of rice when matched to nonparboiled rice.

#### 3.1.7 Energy content

Figure 4a,b shows that the highest energy content is recorded at 60°C of soaking temperature for 4.5–5.5 h for the NERICA‐4 variety. The lowest energy content was recorded between 63 and 70°C of soaking temperature and 5–6 h of soaking time. For NERICA‐6 variety, the highest energy content value was observed between 69 and 70°C of soaking temperature for 4 h, whereas the lowest energy contents were recorded between 60 and 67.5°C for 6 h (Figure 4b). The results showed that the soaking temperature and time improved the energy content of the parboiled rice. The observation agrees with the work of Thomas et al. (2013), who reported that parboiled rice could be considered a good energy source. It could be an excellent parboiled process and the better‐quality raw rice varieties that improve the energy content of brown rice.

### 3.2 Effects of soaking temperature and time on minerals contents of parboiled rice varieties

The impacts of soaking temperature and time on the minerals (magnesium, potassium, and sodium) contents of selected parboiled rice varieties are indicated in Table 3. The results showed that soaking temperature and time had a significant (*p* < .05) impact on the investigated minerals of parboiled rice varieties. The highest value of magnesium (274.72 mg/100 g), potassium (361.24 mg/100 g), and phosphorous (320.053 mg/100 g) contents were recorded in NERICA‐4 soaked at 60°C for 5 h, 65°C for 6 h, and 60°C for 4 h, respectively. At the same time, the lowest values of magnesium (84.70 mg/100 g) and potassium (58.49 mg/100 g) were observed in NERICA‐6 variety soaked at 70°C for 4 h, whereas phosphorus (63.35 mg/100 g) soaked at 60°C for 4 h. The variations might be due to the amount of rice bran removed from the grain during milling, polishing, and leaching out with soaking water during the parboiling process. The findings are similar to the results reported by Ale et al. (2015). The effects of soaking temperature and time on the minerals contents of parboiled rice varieties recorded in this study are in line with the findings of Ale et al. (2015) and Ayamdoo et al. (2015).

**TABLE 3:** Effects of soaking temperature and time on minerals composition of parboiled rice varieties.

Temp (°C)	Time (h)	Varieties	Mg/100 g	K mg/100 g	P mg/100 g
60	6	NERICA‐4	201.62	205.78	275.32
60	4	NERICA‐4	251.55	348.92	109.98
70	6	NERICA‐6	118.42	197.86	92.31
70	4	NERICA‐4	185.94	259.94	83.44
60	5	NERICA‐6	96.84	75.301	178.48
60	4	NERICA‐4	265.58	361.24	106.68
70	4	NERICA‐6	84.72	158.92	106.29
70	4	NERICA‐6	142.86	58.491	127.29
70	4	NERICA‐4	140.26	157.99	112.00
60	4	NERICA‐6	108.94	138.37	63.35
60	5	NERICA‐4	235.73	340.81	320.05
67.5	5	NERICA‐4	205.94	305.61	290.50
70	6	NERICA‐4	264.83	328.45	258.51
70	6	NERICA‐6	118.52	104.38	134.15
65	5	NERICA‐6	156.08	105.99	136.92
60	6	NERICA‐6	161.20	105.47	131.34
65	6	NERICA‐4	274.72	318.35	268.31

Abbreviations: K, Potassium; Mg, magnesium; P, Phosphorus.

### 3.3 Effects of soaking temperature and time on physical properties of parboiled rice varieties

Effects of soaking temperature and time on the physical properties of parboiled rice varieties such as cooking time and hardness are indicated in Table 4.

**TABLE 4:** Mean values of physical properties of parboiled rice varieties.

Soaking temperature (°C)	Soaking time (h)	Variety	Hardness (*N*)	Cooking time (min)
60	6	NERICA‐4	374.09	47
60	4	NERICA‐4	366.68	35
70	6	NERICA‐6	392.01	60
70	4	NERICA‐4	371.88	42
60	5	NERICA‐6	379.65	56
60	4	NERICA‐4	368.31	38
70	4	NERICA‐6	373.30	52
70	4	NERICA‐6	376.27	54
70	4	NERICA‐4	377.96	55
60	4	NERICA‐6	375.85	53
60	5	NERICA‐4	369.79	41
67.5	5	NERICA‐4	385.71	57
70	6	NERICA‐4	392.3	60
70	6	NERICA‐6	387.37	58
65	5	NERICA‐6	375.18	52
60	6	NERICA‐6	370.94	50
65	6	NERICA‐4	375.37	52

#### 3.3.1 Cooking time

The interaction effects of soaking temperature and time on cooking time of NERICA‐4 and ‐6 rice varieties grown in Eastern Ethiopia are shown in Table 4. The lowest cooking time (35 min) was recorded in the NERICA‐4 variety at 60°C soaking temperature with 4 h of soaking time, whereas the highest was observed at 70°C of soaking temperature and 6 h of soaking time in both NERICA‐4 and ‐6 varieties. The results showed that the cooking time increased as the soaking temperature and soaking time were increased. The found results in contrast with Meresa et al. (2020) studied that the minimum cooking times of Ethiopian rice varieties were identified as 16–23 min for Ediget and 16–23 min for Gumara rice varieties when soaked at 40–80°C for 10–50 min. The variation in cooking time might be due to differences in genetic makeup and microwave‐assisted parboiling equipment which are significantly decreased the cooking time of the rice grains (Rockembach et al., 2019).

**FIGURE 5:**
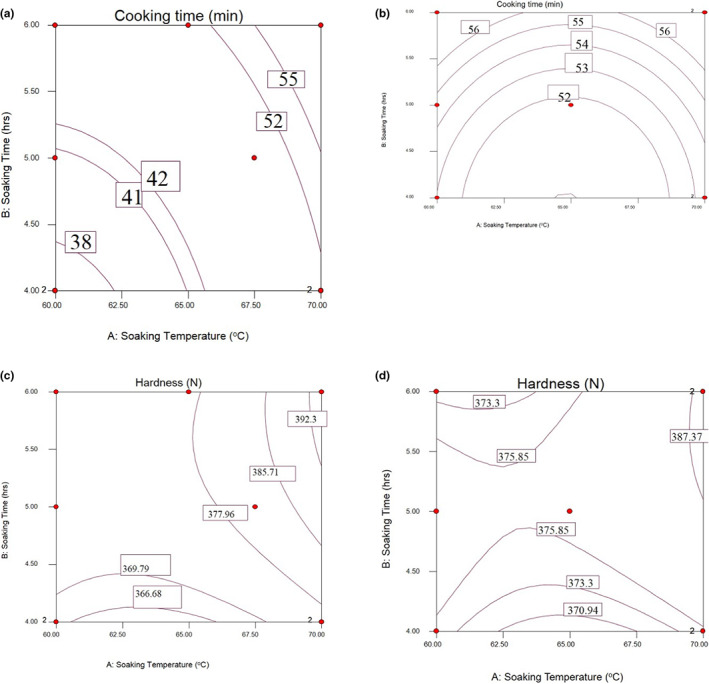
Response surfaces for cooking time of NERICA‐4 (a), the cooking time of NERICA‐6 (b), the hardness of NERICA‐4 (c), and the hardness of NERICA‐6 (d) of parboiled rice kernels.

According to results presented in Figure 5a,b, lower cooking time between 60 and 62.25°C of soaking temperature for 4 h. The highest cooking time was observed in 65–70°C of soaking temperature and 5–6 h of soaking time for NERICA‐4 variety. For NERICA‐6, the lower cooking time was recorded between 60.85 and 69°C of soaking temperature for 4 h. The highest cooking time was recorded between soaking temperatures of 60–70°C and soaking time of 5 h. It could be related to less removal of the bran layer of brown rice, which allowed the rice to absorb more water which causes lower moisture diffusion and higher water absorption. This, in turn, reduced the internal void space of starchy endosperm. The observation in this study agreed with the findings of Mohorič et al. (2009). The endosperm of the parboiled rice has a more compressed starch structure, and the decreased porosity tends to increase the cooking time. The variation might also have occurred due to the global rice varieties, storage duration, and way processing parboiling conditions (soaking temperature and time). This may affect the cooking time described by Mo et al. (2019). It may be related to less removal of the bran layer of brown rice, prohibiting the rice from obtaining more water (lower moisture diffusion).

#### 3.3.2 Hardness

Lower hardness was recorded between 60.5 and 66°C of soaking temperature at 4 h soaking time, whereas the highest hardness value was recorded between 68.5 and 70°C and 5.5–6 h (Figure 5c). For NERICA‐6 variety, the minimum hardness value was obtained between 62 and 67°C of soaking temperature for 4 h. The highest hardness value was recorded between 68 and 70°C of soaking temperatures for 5–6 h (Figure 5d). The result was similar to Kale et al. (2017). They reported that higher soaking temperatures imparted more hardness to the rice kernel. This study confirmed that soaking temperature increases as soaking time increases and rice varieties affect the hardness. The found result agreed with the work of Corrêa et al. (2007). Parboiling fills the void spaces and cement cracks, reducing porosity inside the endosperm and making the grain harder.

### 3.4 Optimization of soaking temperature and time for parboiled rice

The optimization goal was to get optimum parboiling conditions for better physical–chemical properties. It was carried out by setting criteria (minimum, maximum, and range) for each response to find the optimum level of independent variables (soaking time and temperature) that could produce the best quality parboiled for both NERICA‐4 and NERICA‐6, as indicated in Table 5. Accordingly, a soaking temperature of 65°C and soaking time of 6 h were found to be the optimum value for hardness (375.37 N), cooking time of 52 min, moisture 12.3, ash (1.24%), protein (13.86%), fat (2.17%), fiber (3.29%), carbohydrate (67.11%), gross energy (343.5 kcal/100 g), magnesium (274.72 mg/100 g), potassium (318.35 mg/100 g), and phosphorous (268.31 mg/100 g) for NARICA‐4, whereas the optimum soaking temperature and time obtained for NARICA‐6 were 65°C and 5 h in which hardness cooking time, moisture 12.2, ash (1.4%), protein (11.54%), fat (2.29%), fiber (2.89%), carbohydrate (69.6%), gross energy (345.42 kcal/100 g), magnesium (156 mg/100 g), potassium (105.9 mg/100 g), and phosphorous (136.9 mg/100 g, respectively), 375.18 N, 52 min, 12.2%, 1.4%, 11.54%, 2.29%, 2.89%, 69.6%, 345.42 kcal/100 g, 156 mg/100 g, 105.9 mg/100 g, and 136.9 mg/100 g, respectively.

**TABLE 5:** Optimization responses of physicochemical properties for values of parboiling conditions.

Name	Goal	Lower limit	Upper limit	Optimum value obtained NERICA‐4	Optimum value obtained NERICA‐6
Hardness (*N*)	Minimize	366.68	392.30	375.37	375.18
Cooking time (min)	Minimize	35.00	60.00	52.00	52.00
Moisture %	Minimum	10.70	14.50	12.30	12.20
Ash %	Maximize	0.80	2.04	1.24	1.40
Protein %	Maximum	9.924	13.86	13.86	11.54
Fat %	Maximize	1.66	3.52	2.17	2.29
Fiber %	Maximize	1.73	3.29	3.29	2.89
Carbohydrate %	Maximize	67.11	72.27	67.11	69.60
Energy kcal/100 g	Maximize	335.72	358.69	343.50	345.42
Magnesium mg/100 g	Maximize	84.72	274.72	274.72	156.00
Potassium mg/100 g	Maximize	58.49	361.24	318.35	105.90
Phosphorus mg/100 g	Maximize	63.35	320.05	268.31	136.90

## 4 CONCLUSION

This study reported optimization of soaking conditions (temperature and time) on physicochemical properties of selected parboiled rice varieties grown in Eastern Ethiopia. The findings showed that soaking time and temperature significantly (*p* < .05) affected the physicochemical properties of both rice varieties. The benefits of parboiling two types of rice include improving the nutritional content and significance of the existence of various phytochemicals, such as fiber, minerals, and nutritional value like protein, and fats. Numerical optimization results generated better rice in terms of physicochemical responses at a soaking temperature of 65°C and soaking time of 6 h for NARICA‐4, and soaking temperature of 65°C and soaking time of 6 h for NARICA‐6 with improvements in ash, protein, fat, fiber, carbohydrate, energy, magnesium, and potassium and phosphorous contents while significant reduction of hardness, cooking time and moisture contents. Generally, the findings suggested that NARICA‐4 rice variety is better in physical properties, proximate composition, and mineral content than NARICA‐6 variety.

## FUNDING INFORMATION

The work is not financially supported.

## CONFLICT OF INTEREST STATEMENT

There are no conflict of interest declared by the authors.

## Data Availability

The data that support the findings of this study are available from the corresponding author upon reasonable request.
